# Deletion of *ssnA* Attenuates the Pathogenicity of *Streptococcus suis* and Confers Protection against Serovar 2 Strain Challenge

**DOI:** 10.1371/journal.pone.0169791

**Published:** 2017-01-12

**Authors:** Miao Li, Ru-Jian Cai, Chun-Ling Li, Shuai Song, Yan Li, Zhi-Yong Jiang, Dong-Xia Yang

**Affiliations:** 1 Institute of Animal Health, Guangdong Academy of Agricultural Sciences, Guangzhou, China; 2 Guangdong Open Laboratory of Veterinary Public Health, Guangzhou, China; 3 Guangdong Provincial Key Laboratory of Livestock Disease Prevention, Guangzhou, China; Instituto Butantan, BRAZIL

## Abstract

*Streptococcus suis* serotype 2 (SS2) is a major porcine and human pathogen which causes arthritis, meningitis, and septicemia. *Streptococcus suis* nuclease A (SsnA) is a recently discovered deoxyribonuclease (DNase), which has been demonstrated to contribute to escape killing in neutrophil extracellular traps (NETs). To further determine the effects of *ssnA* on virulence, the *ssnA* deletion mutant (Δ*ssnA*) and its complemented strain (C-Δ*ssnA*) were constructed. The ability of Δ*ssnA* mutant to interact with human laryngeal epithelial cell (Hep-2) was evaluated and it exhibited dramatically decreased ability to adhere to and invade Hep-2 cells. This mutation was found to exhibit significant attenuation of virulence when evaluated in CD1 mice, suggesting *ssnA* plays a critical role in the pathogenesis of SS2. Finally, we found that immunization with the Δ*ssnA* mutant triggered both antibody responses and cell-mediated immunity, and conferred 80% protection against virulent SS2 challenge in mice. Taken together, our results suggest that Δ*ssnA* represents an attractive candidate for designing an attenuated live vaccine against SS2.

## Introduction

*Streptococcus suis* (*S*. *suis*) is a major swine pathogen responsible for a huge economic cost in the worldwide pig industry [1]. It is also a severe threat to human health by causing severe systemic infection [2–3]. Based on the capsular polysaccharide, 35 *S*. *suis* serotypes have been described. *S*. *suis* serotype 2 (SS2) is considered an emerging zoonotic agent, causing a variety of diseases which include arthritis, meningitis, septicemia, and even acute death in pigs and humans [4–5]. The first case of *S*. *suis*-caused human infection was diagnosed in Denmark in 1968 [6]. So far, SS2 has been reported in more than 20 countries and caused approximately 700 human infections [7–8]. In particular, two large outbreaks have taken place in China in 1998 and 2005 [9–10]. Despite various virulence factors of *S*. *suis* have been identified, limited knowledge on the pathogenesis of *S*. *suis* impedes the attempts to control this organism [11]. *S*. *suis* infection remains a serious infectious disease challenge to global public health and food safety. Therefore, there needs to carry out more researches to identify new virulence factors and understand *S*. *suis* infection mechanism.

It is known that many pathogenic bacteria produce secreted deoxyribonucleases (DNases), which are regarded as virulence factors [12–13]. More specifically, DNases have been shown to be involved in biofilm maturation and bacterial growth [14–15]. Recent studies implied that secreted DNases contribute to pathogenesis by enhancing evasion of the innate immune response [13, 16]. *Streptococcus suis* nuclease A (SsnA) is a recently discovered cell wall—anchored extracellular DNase, which present in most of the serotypes and clinical isolates analyzed. The study has confirmed that the *ssnA* gene is expressed during all the growth process of *S*. *suis*, and reacts with *S*. *suis* immune sera confirmed that SsnA is expressed *in vivo* [17]. SsnA could be a good vaccine formulation against *S*. *suis*, as it is well exposed and approachable to antibodies, being also very immunogenic [18–19]. Although the characterization of DNase activity of *ssnA* and its immunogenicity have been previously reported, the actual contribution of this DNase on *S*. *suis* virulence remains to be verified.

In this study, an attenuated SS2 strain was developed by deleting the *ssnA* gene of the GD01 strain. Our results showed that *ssnA* is a pathogenicity-associated determinant of *S*. *suis* involved in growth, adhesion and invasion to host cell. The Δ*ssnA* mutant represents a promising vaccine candidate of SS2 with comparable safety in murine animal models.

## Materials and Methods

### Ethics statement

All animal procedures were approved by the Ethics Committee of Institute of Animal Health, Guangdong Academy of Agricultural Sciences according to Guangdong Province Laboratory Animal Management Regulations—2010. The license number was SYXK(Yue) 2011–0116. All efforts were made to minimize suffering. Humane endpoints used during the animal survival study were: rapid weight loss of >20% of body weight, poor physical appearance (reduced mobility, rough coat and depression), rapid breathing, swollen eyes, and joint tumefaction. Following infection, the healthy status of animals was evaluated every 8 h and there were not unexpected deaths. Animals that reached humane endpoints were euthanized through complete exsanguination via cardiac puncture under general anesthesia with inhaled 2% isoflurane.

### Bacterial strains, culture conditions, and plasmids

The bacterial strains and plasmids used in this study are describes in Table 1. SS2 wild-type (WT) strain GD01 was isolated from the brain of a sick pig in Guangdong, China. *S*. *suis* was grown in tryptic soy broth (TSB) or plated on tryptic soy agar (TSA) plates (Difco, Detroit, MI, USA) plus 5% bovine sera at 37°C. *E*. *coli* strains were cultured in Luria-Bertani (LB) broth or on LB agar plates. Antibiotics were used as follow: 100 μg/mL of spectinomycin (Spc), 5 μg/mL of chloramphenicol (Cm), or 8 μg/mL of erythromycin (Erm).

**Table 1:** Bacterial strains and plasmids used in this study.

Strains/plasmid	Characteristics/Functions	Source/Reference
Bacterial strains		
*E*. *coli* DH5a	Cloning vehicle: clone the upstream and downstream flanking regions of *ssnA*	Invitrogen
*E*. *coli* BL21(DE3)	For expressing the recombinant plasmids	Invitrogen
*S*. *suis* GD01	Serotype 2, clinical isolated virulent strain	Laboratory collection
Δ*ssnA*	The deletion mutant of *ssnA* with background of GD01	This study
C-Δ*ssnA*	Complemented strain of *ssnA*	This study
Plasmids		
pMD19-T	Cloning vector; Amp^R^	TaKaRa
pSET4s	*E*. *coli*–*S*. *suis* shuttle vector thermosensitive suicide; Spc^R^	Takamatsu et al., 2001
pSET4sΔ*ssnA*	A recombinant vector with the background of pSET4s, designed to knockout *ssnA*; Spc^R^	This study
pAT18	A plasmid carrying an erythromycin resistance rRNA methylase (Erm) gene expression cassette	Trieu-Cuot et al., 1991
pAT18-*ssnA*	A recombinant vector with the background of pAT18, designed to complementation *ssnA*; Erm^R^	This study

### Construction of the mutant and functional complementation strains

Primers used for amplification (Table 2) in this study were ordered from the Invitrogen (Shanghai, China). The thermosensitive plasmid pSET4s was used for the substitution of the *ssnA* with the *Cm* in *S*. *suis* strain. The upstream (865 bp) and downstream (624 bp) fragments flanking regions of *ssnA* were amplified from SS2 WT strain GD01 genomic DNA using PCR with two pairs of primers: P1-F/P1-R and P2-F/P2-R. The *Cm* was amplifed with specific primer Cm-F/Cm-R. The PCR products were digested using the corresponding restriction enzymes and inserted between the *Sal* I and *Sca* I site of pSET4s to generate the recombinant plasmid pSET4sΔ*ssnA*. This plasmid was confirmed by DNA sequencing and restriction analysis, and then was electrotransformed into GD01 strain. The temperature sensitive replication of this vector enabled allelic replacement of *ssnA* with *Cm* in the SS2 chromosome to generate the mutant strain Δ*ssnA* [20]. Allelic exchange mutagenesis of *ssnA* was confirmed by PCR with four primer pairs (SS-16S-F/SS-16S-R, Cm-F/Cm-R, Spc-F/Spc-R, and P3-F/P3-R) and sequence analysis.

**Table 2:** Oligonucleotide primers used in this study.

Primers	Primers sequence (5’-3’)*	Amplification for
P1-F	gagtcgactcaaactgtctgggaga	Upstream border of *ssnA*
P1-R	gcggatccataaaactcctttttc	
P2-F	gaggatcctcattttttgagcttg	Downstream border of *ssnA*
P2-R	gcgagctcgctccaggtaaaactt	
Cm-F	gcggatcctaattcgatgggttccgagg	Cm^R^ expression cassette
Cm-R	tctggatccgaaaacactagagcttgatg	
Spc-F	gtgttcgtgaatacatgttata	Spc^R^ expression cassette
Spc-R	gttttctaaaatctgattacca	
P3-F	gttaaacaaaatgaatatggcacc	the part of upstream homologous arm region, the intermediate region, and the part of down homologous arm region of *ssnA*
P3-R	attgacagccaaaatctgattggc	
SS-16S-F	cagtatttaccgcatggtagatat	*S*.*suis* 16S rRNA
SS-16S-R	gtaagataccgtcaagtgagaa	
P4-F	gcggaattctgttaaacaaaatgaatat	*ssnA* coding sequence and its promoter
P4-R	gcggatccttaggattctttttg	

* Underlined nucleotides denote enzyme restriction sites.

To obtain a complementary strain, the fragment containing the *ssnA* gene and its upstream promoter was amplified with primer pair (P4-F and P4-R), and then was cloned into the shuttle vector pAT18 [21] to generate the plasmid pAT18-*ssnA*. This plasmid was introduced into Δ*ssnA* to obtain the function complemented strain C-Δ*ssnA* by Erm resistance screening in TSA plates.

### Growth characteristic

The SS2 WT GD01, Δ*ssnA*, and C-Δ*ssnA* strains were separately inoculated in 5 mL TSB containing 5% bovine sera, and incubated at 37°C for 12 h. Then the cultures were diluted to the optical density at 600 nm (OD_600_) of 0.6 and inoculated into 100 mL TSB. OD_600_ values for three cultures were determined using spectrophotometer (Bio-Rad, Hercules, CA, USA) at 1 h intervals. Uninoculated media served as the blank control.

### Cell adhesion and invasion assays

The human laryngeal epithelial cell (Hep-2) line was cultivated as previously described [22]. Briefly, cells were maintained as a monolayer in 24-well tissue culture plates. *S*. *suis* cultures (WT, Δ*ssnA*, and C-Δ*ssnA*) were pelleted and washed three times with PBS (pH 7.4). Cells were infected with 10^6^ CFU/mL bacterial suspension at 37°C with 5% CO_2_ for 2 h. Then the plates were rinsed with PBS, and 100 μL PBS containing 0.25% trypsin/0.02% EDTA was added to lyse cells at 37°C for 10 min. The lysates were diluted 10-fold and placed onto TSA agar to calculate viable bacteria.

For the invasion assay, the minimal inhibitory concentration (MIC) values of three strains were determined as the lowest concentration of antimicrobial agent that prevented visible growth. Then 100 μg/mL gentamycin (MIC = 16 μg/mL) was added to the Hep-2 cells to kill non-invaded bacteria, prior to lysing the cells.

### Effect of *ssnA* on virulence in CD1 mice

The virulence of the WT GD01, mutant strain Δ*ssnA*, and C-Δ*ssnA* were determined by 50% lethal dose (LD_50_) value. Ninety-six six-week-old female CD1 mice were randomly divided into 16 groups. The WT, Δ*ssnA*, and C-Δ*ssnA* strains were cultured at 37°C and then diluted to appropriate concentration. The mice were infected intraperitoneally (i.p.) with 500 μL of the bacterial suspension (Table 3). The mice injected only with PBS were served as negative controls. The mortality of mice was recorded for 7 days, and the LD_50_ values were calculated using the method of Karber [23].

**Table 3:** Determination of LD_50_ in CD1 mice infected with the WT GD01, Δ*ssnA*, and C-Δ*ssnA* strains.

Strains	Challenge dose (CFU)	Number dead	Value of LD_50_ (CFU)	Fold attenuationc ^a^
	1.3×10^7^	0		
	2.0×10^7^	1		
Wild-type (GD01)	4.0×10^7^	3	4.42×10^7^	1
	8.0×10^7^	4		
	1.3×10^8^	6		
	2.0×10^8^	1		
	4.0×10^8^	2		
Mutant (Δ*ssnA)*	8.0×10^8^	4	6.17×10^8^	14
	1.6×10^9^	4		
	3.2×10^9^	6		
	4.0×10^7^	1		
	8.0×10^7^	2		
C-Δ*ssnA*	1.2×10^8^	4	7.63×10^7^	2
	1.6×10^8^	6		
	2.0×10^8^	6		

^a^ Fold attenuation normalised to the wild-type strain.

### Determination of viable bacteria invasion in susceptible tissues

Thirty female CD1 mice were classified into three groups and injected i.p. with 500 μL of GD01, Δ*ssnA*, or C-Δ*ssnA* (5 × 10^6^ CFU). Five mice inoculated with PBS were served as controls. One control and three infected mice were euthanased at 3, 5, and 7 day post-infection to assess the bacterial invasion of WT, Δ*ssnA*, or C-Δ*ssnA* strains in infected tissues. Blood samples (10 μL) collected from the tail vein and homogenized spleen, liver, and brain samples (0.05 g/ tissue) were plated on TSA plates.

### Competitive-infection assay

A 1:1 mixture of GD01 and Δ*ssnA* in 200 μL TSB was inoculated i.p. into six mice. After inoculation for 8 h, the blood samples from the infected mice were collected and diluted to plate onto TSA plates. The competitive index (CI) was calculated as follow: CI = [CFU of mutant strain in blood/CFU of WT strain in blood] / [CFU of mutant strain in original inoculum/CFU of WT strain in original inoculum].

### Immunization and challenge

The protection assay was carried out in the CD1 mouse model. Forty-five six-week-old female CD1 mice were randomly assigned to 3 groups of 15 each. Group 1 were immunized with 10^8^ CFU of Δ*ssnA* in 200 μL PBS. Group 2 were immunized with the SS2 inactivated vaccine, and Group 3 were injected with 200 μL of PBS served as a negative control. All animals were vaccinated twice with 2-week interval.

At 28 days post-immunization, blood samples were obtained by tail vein bleeding, and then all animals were challenged i.p. with 6.0×10^9^ CFU (5 × LD_50_) of GD01 in 500 μL of PBS. At 14 days post-challenge, the surviving mice were euthanized.

### Determination of antibody levels

The antigen-specific serum IgG levels were detected using an indirect enzyme-linked immunosorbent assay (ELISA). Briefly, microtitre plates were coated with 5 μg/100 μL of the killed bacteria SS2 at 4°C overnight. Following washing and blocking, serial dilutions of sera were added and incubated for 2 h at 37°C. After washing five times, the plates were added with anti-mouse IgG (1: 5000) horseradish peroxidase (HRP)-conjugated antibodies (Sigma, USA) for 1 h at 37°C. Upon washing, 100 μL/well of TMB was added for color reaction. The reaction was stopped by adding 50 μL 2 M H_2_SO_4_ to each well. OD_450 nm_ was measured using an ELISA plate reader (Bio-Rad, USA).

### Lymphocyte proliferation

On 14 days post the second immunization, three mice from each group were sacrificed and spleens were isolated under sterile condition. Splenocyte pellets were collected by centrifuge and resuspended in complete DMEM medium (10^5^ cells/mL) (Hyclone, Logan, UT, USA). Lymphocytes were cultured in 96 well culture plates and incubated with the heat-killed SS2 strain (5 × 10^8^ CFU/mL) for 72 h at 37°C with 5% CO_2_. Cultures were made in medium along as a negative control, or containing 5 μg/well of concanavalin A (ConA) (Sigma, USA) as a positive control. Lymphoproliferation assays were carried out with MTT reagent using a lymphocyte proliferation kit (Promega, USA) following the manufacturer’s protocol. The absorbance at 490 nm was measured using an ELISA reader.

### Cytokines detection assay

Quantitative analyses of interleukins (IL)-10, IL-4, IL-2, and interferon(IFN)-γ were performed with cytokine detection kits, following manufacturer’s instructions (R & D, Minneapolis, MN, USA).

### Statistical analysis

The data were analyzed with Excel software and presented as the mean ± SD. One-way analysis of variance followed by Student’s t-test was used to analyze the difference between two groups. Differences among three groups were analyzed using ANOVA. Statistical significance was defined at *p* < 0.05, and highly statistical significance was defined at *p* < 0.01.

## Results

### Construction of Δ*ssnA* mutant strain and complemented strain

**Fig 1:**
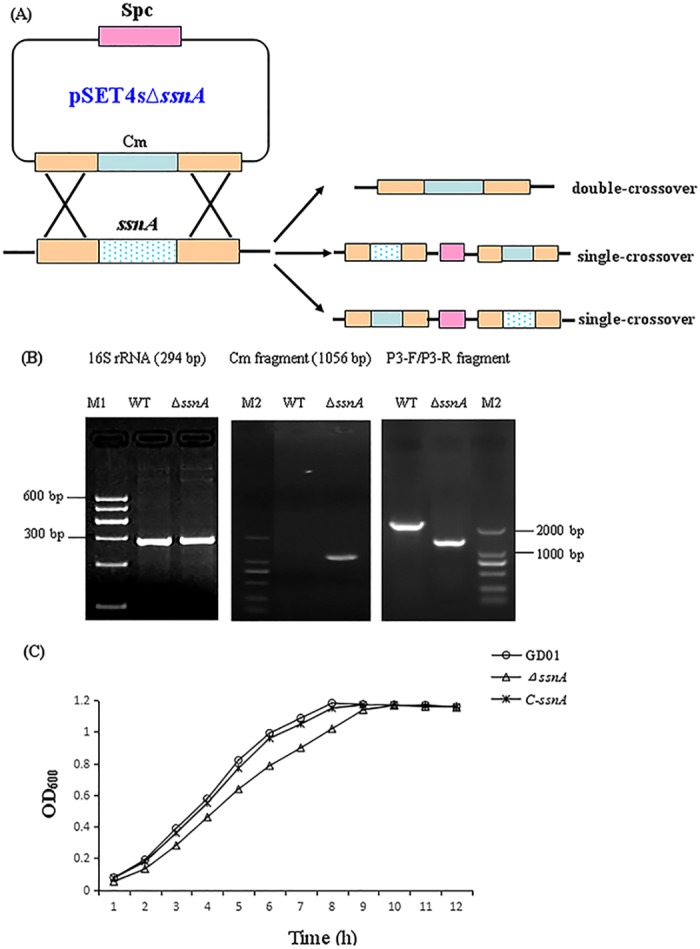
Construction of the Δs*snA* mutant strain and complementation strain. (A) Schematic of the strategy for the *ssnA* gene deletion in the SS2 GD01 strain via double-crossover recombination. The plasmid pSET4sΔ*ssnA* is used for the *ssnA* gene knockout. (B) PCR confirmation of the mutant strain. (C) Growth of the WT GD01, Δ*ssnA* mutant, and its complementation.

In order to determine whether the function of the *ssnA* was crucial for virulence in *S*. *suis*, we deleted the *ssnA* gene of SS2 GD01 strain using a double-crossover homologous recombination approach (Fig 1A). The deletion of the *ssnA* was confirmed by DNA sequencing (data not shown) and PCR (Fig 1B), verifying that the *ssnA* mutant was successfully constructed. The *S*. *suis* 16S rRNA fragment (294 bp) was detectable in both of the WT and mutant strains. The primer P3-F/P3-R amplified a bigger band in the WT strain, because the sequence of *ssnA* is longer than that of the Cm resistance cassette. The primers Cm-F/Cm-R amplified a 1056 bp fragment in the Δ*ssnA* strain, while did not obtain a band from WT strain. The Spc fragment was undetectable in both of strains (data not shown). The complemented strain construction method was transformation of GD01 with plasmid pAT18-*ssnA*, and C-Δ*ssnA* was identified by Erm-resistance screening.

### *SsnA* is required for growth of SS2

Growth curves of the WT, Δ*ssnA* mutant, and C-Δ*ssnA* strains were determined at OD_600_, which showed that Δ*ssnA* exhibited slightly lower growth than the WT and C-Δ*ssnA* in the early-exponential phase and mid-exponential phase (Fig 1C). This result indicated that *ssnA* plays a role in the growth of SS2.

### The *ssnA* of SS2 contributes to the adherence to and invasion of Hep-2 cells

**Fig 2:**
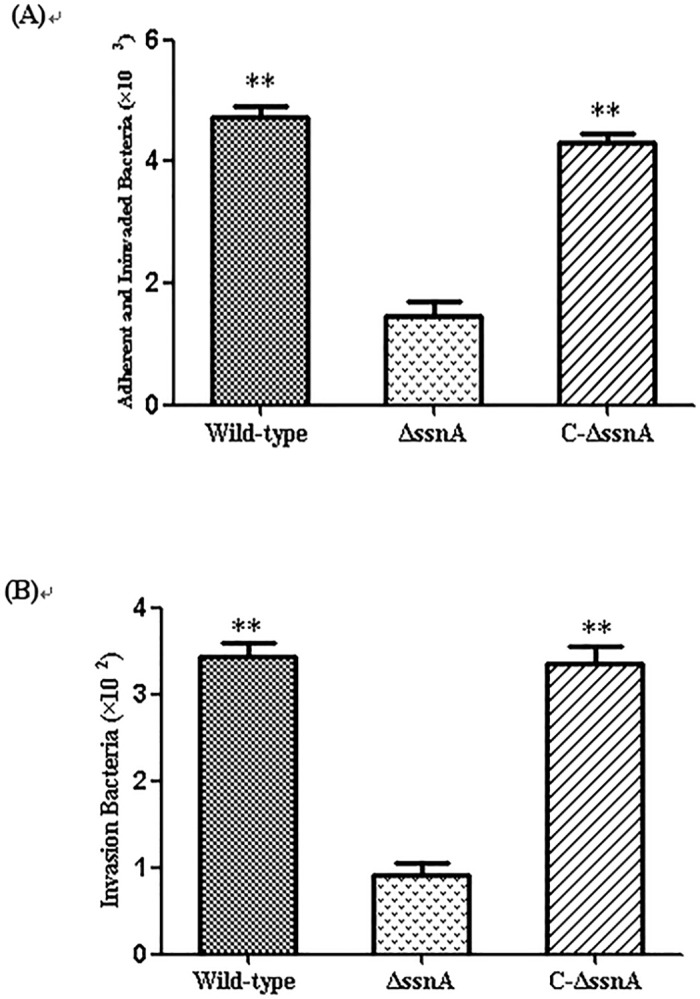
The WT, Δ*ssnA*, and C-Δ*ssnA* strains adhere to and invade (A), and invasion of (B) Hep-2 cells after 2 h of infection. The data are means and SD of three independent experiments, **P < 0.01.

To check whether the *ssnA* interacted with host cells, we used Hep-2 cells to compare the adherence and invasion abilities in the WT GD01, Δ*ssnA* mutant, and complementation strains (Fig 2). In the adhesion assay, the numbers of adhesion bacterial in the extracellular and invasion to the intracellular were detected (Fig 2A). After 2 h of infection, the Δ*ssnA* mutant exhibited the lowest level of adherence. Compared to the Δ*ssnA* strain, the WT strain showed significantly increased adherence capability in Hep-2 cells (*p* < 0.01), whereas the level of adhesion was fully recovered in the complemented strain. The similar results were obtained in the invasion assay (Fig 2B).

### The absence of *ssnA* affects SS2 virulence in mice

To evaluate the effect of *ssnA* on virulence, the CD1 mice were injected with various doses of GD01, Δ*ssnA*, or C-Δ*ssnA*. In the higher inoculum dose groups, infected mice showed obvious clinical signs of SS2 infection, including depression, irregular coat, swollen eyes, and rapid breathing. As shown in Table 3, the LD_50_ was 4.42 × 10^7^, 6.17 × 10^8^, and 7.63× 10^7^ CFU for the WT, Δ*ssnA*, and C-Δ*ssnA* strains, respectively. Mutant Δ*ssnA* was attenuated by 14-fold compared with the WT, indicating that *ssnA* deletion significantly reduced the virulence of SS2.

### Invasion ability of Δ*ssnA* in susceptible tissues

**Fig 3:**
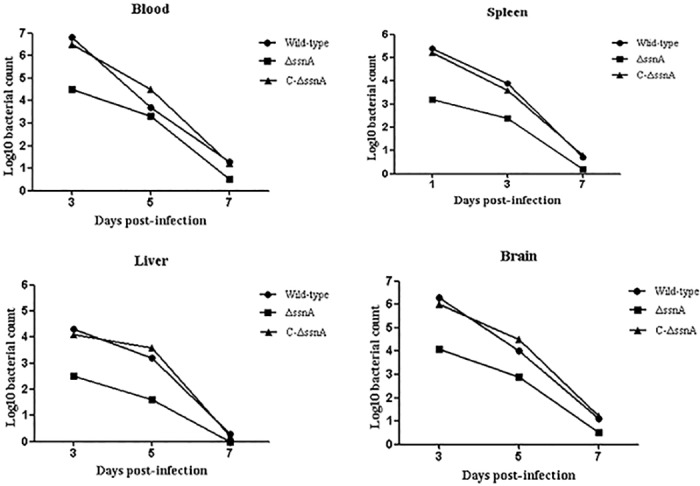
Detection of the WT, Δ*ssnA*, and C-Δ*ssnA* strains invasion in blood and tissues. Blood (A), spleens (B), livers (C), and brains (D) were collected from infected CD1 mice and homogenized at 3, 5, and 7 days after infection.

**Fig 4:**
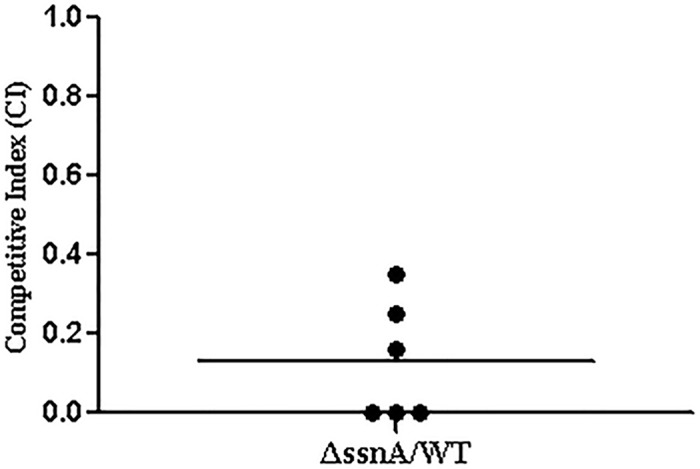
The CI of Δ*ssnA* against the WT strain *in vivo*. Six CD1 mice were infected i.p. with Δ*ssnA*: WT at 1:1 ratio. At 8 h post inoculation, the value of CI in blood samples was calculated.

To analyze the virulence attenuation conferred by Δ*ssnA*, the invasion experiments *in vivo* were performed. The mice were infected with 5 × 10^6^ CFU of the WT, Δ*ssnA*, or complementation strain according to the LD_50_ assessment results. At different time-points, the viable bacteria were recovered from liver, spleen, brain, and blood. Compared with the WT-infected mice, bacterial counts in blood and organs of the Δ*ssnA*-infected mice were significantly decreased (Fig 3). Furthermore, the abilities of the WT and Δ*ssnA* strains to colonize the blood were evaluated by competitive-infection assay. Six mice were infected with the WT and Δ*ssnA* in a 1:1 ratio. The CI value showed that the CFU of the mutant strain *in vivo* were significantly lower than that of the WT strain (Fig 4), indicating the Δ*ssnA* had a decreased ability of invasion in blood. Taken together, above results suggested that *ssnA* is important for the pathogenesis of SS2.

### Evaluation of immune responses conferred by the Δ*ssnA* mutant

**Fig 5:**
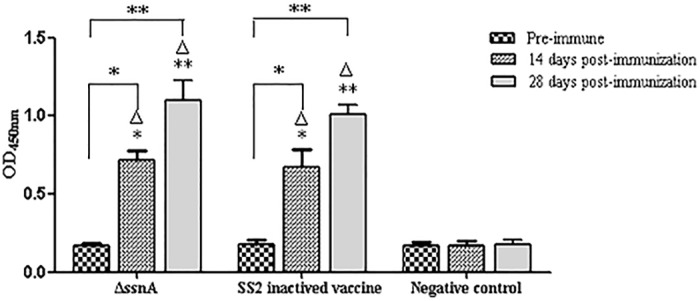
Production of SS2-specific IgG antibodies in immunized mice. The CD1 mice were immunized with SS2 inactive vaccine, Δ*ssnA*, or PBS, and antibody levels were detected at 14, and 28 days post-inoculation by indirect ELISA. The data are expressed as the mean ± SD, *P < 0.05 and **P < 0.01. Δ, versus the negative control.

Serum samples were collected from each group at 14 and 28 day post-immunization and the humoral responses induced by Δ*ssnA* were detected by indirect ELISA. As shown in Fig 5, the mice vaccinated with the mutant and SS2 inactivated vaccine exhibited significant IgG antibody response (*p* < 0.01), whereas no specific serum IgG antibody against bacteria antigen were detected in the PBS group.

**Fig 6:**
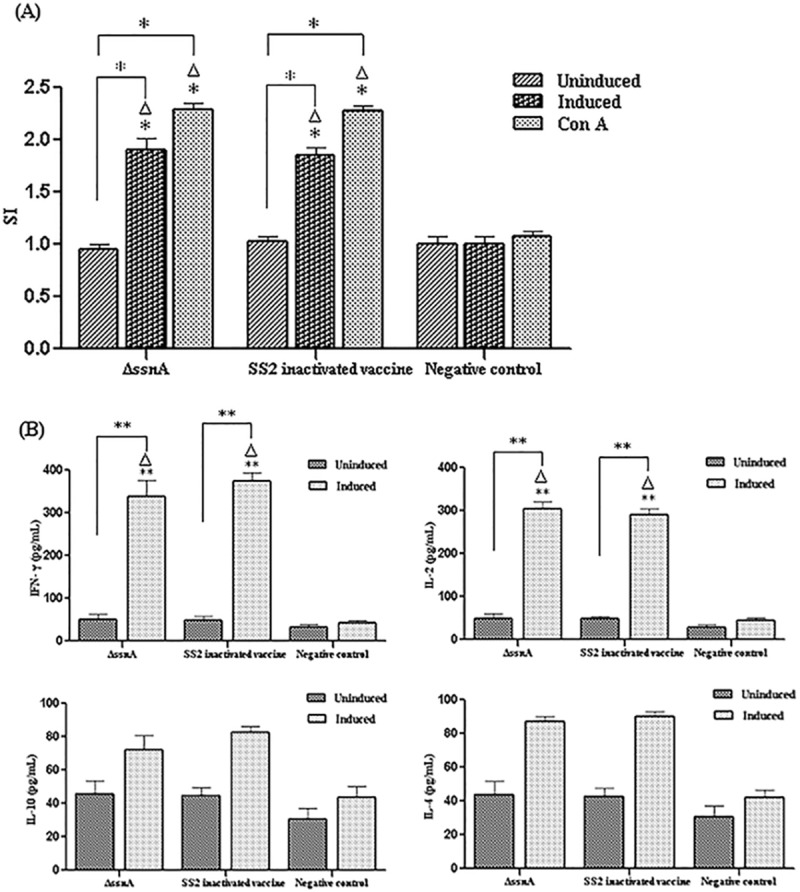
The cellular immune responses induced in immunized mice. (A) Splenocytes from immunized mice were collected at 28 day post-first immunization and lymphocyte proliferation assay was evaluated using a cell proliferation kit with MTT reagent. (B) Production of cytokines in stimulated splenocytes was detected by ELISA. *P < 0.05. Δ, versus the negative control.

At 28 day post-inoculation, splenocytes supernatants collected from immunized mice were assessed for lymphocyte proliferation to characterize the cellular immune response (Fig 6A). The splenocytes from both immunized groups stimulated with ConA and heat-killed *S*. *suis* strain produced significant proliferative T-cell immune response (*p* < 0.05), whereas no proliferative response in the negative control group.

The levels of cytokines of splenocytes stimulated with either *S*. *suis* strain or medium alone are presented in Fig 6B. The Th1-type cytokine IFN- γ and IL-2 and the Th2-type cytokine IL-4 and IL-10 were assessed. Compared with the control group, the levels of IFN- γ and IL-2 were significantly high in the vaccine immunized groups (*p* < 0.01), which significantly greater than the IL-4 and IL-10 levels.

All above results suggested that the *ssnA* mutant induced both antibody and cell-mediated immunity with a dominant Th1 immune response.

### Protective efficacy against SS2 challenge in mice

**Fig 7:**
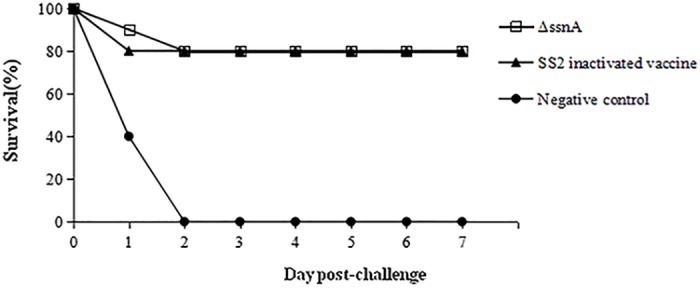
Survival curves of mice challenged with a high-virulent SS2 strain.

To scrutinize the ability of the Δ*ssnA* to protect against virulent SS2, the challenge study was performed and clinical symptoms of *S*. *suis* infection and mortality were observed. Compared with the negative group, the Δ*ssnA-*vaccinated mice were significantly protected against SS2 challenge (Fig 7). All mice from control group showed obvious clinical signs, while in the vaccinated groups there were only a few mice showed temporal and slight depression. The Δ*ssnA* mutant induced 80% protection against challenge with a dosage of 5-fold of the LD_50_ of WT SS2 in mice.

## Discussion

DNases are common among prokaryotes. Intracellular bacterial DNases participate in replication, recombination or DNA repair to maintain chromosome and plasmid integrity. In contrast, extracellular DNases have been implicated as virulence factors in several species [24–26]. Nuclease activity in *S*. *suis* was first reported in 2004 [17]. The *ssnA* gene encoded a cell-associated DNase has been identified in SS2. Previous study has indicated that the amino-acid sequence of SsnA is high homology to the SpnA protein of *Streptococcus pyogenes*, which is a novel virulence factor of group A *streptococcal* [13]. Recent research has showed that SsnA is the first specific factor of neutrophil extracellular trap (NET) evasion [27], but the functional roles of this gene on virulence and immunogenicity were unknown. In present study, the *ssnA* mutant GD01Δ*ssnA* and its complementation strain were constructed to investigate the effects of *ssnA* gene-deletion in *S*. *suis* and evaluate the potential of Δ*ssnA* for developing attenuated vaccine. Based on the result of growth curves of the WT GD01, Δ*ssnA* mutant, and C-*ssnA* strains, we found that the deletion of the *ssnA* has an effect on the growth of SS2 strain. Furthermore, we analyzed the role of *ssnA* in the process of SS2 infection in *vitro* and *vivo*.

Bacterial adherence to host cells is an important step toward cellular invasion, which conduce to break through the cell barrier, persistence, and penetration into deep tissues of the host [28]. The adhesion effect of *S*. *suis* on host epithelial cell is concerned with its invasion in the respiratory and organs of the infected host. Systemic infection of *S*. *suis* requires the circulating bacteria to effectively invade into different tissues and cells. In this study, we have demonstrated that Δ*ssnA* has a markedly impaired interaction with Hep-2 *in vitro*. These findings provide evidence that *ssnA* may be associated with pathogenicity of *S*. *suis*.

To clarify the effect of *ssnA* on virulence, the infection experiment *in vivo* with the mice were performed. We chose the CD1 mouse as animal model for *S*. *suis* infections because it is has been confirmed effective and excellent [29]. In this study, comparing with the WT-infected group, the LD_50_ of Δ*ssnA* mutant was significantly increased. Our results indicated that the *ssnA* gene play an important role on *S*. *suis* virulence. Bacteria invasion experiments *in vivo* were carried out to investigate the virulence attenuation of Δ*ssnA*. A distinct difference of bacterial loads were detected from blood and the tissues of Δ*ssnA-*infected mice compared to those of the mice infected with the WT strain. Interestingly, the clearance rate between the WT and mutant groups were no significant difference. Considering above results, the difference between WT and Δ*ssnA* may come from the different invasiveness from abdominal cavity to blood. These indicated that Δ*ssnA* is required during invasion *in vivo*, but not for bacterial survival *in vivo*. The role of *ssnA* on virulence were further investigated by infecting mice with the WT and mutant strain simultaneously to determine the CI, which is the ratio of bacteria CFU recovered from an individual strain in blood and determines the relative fitness of each strain to survive [30].

Compared with vaccines based on an inactivated bacteria or recombinant protein, attenuated live vaccines are advantageous because they are able to induce good immunity and confer long-term protection. Therefore, we evaluated the attenuated live vaccine potential of the *S*. *suis* Δ*ssnA* mutant. The mice vaccinated with the Δ*ssnA* strain could elicit a high IgG level. In general, vaccine antigen induced antibody level is usually in accordance with its protection rate. In the later challenge study, the mice from two vaccines immunized groups showed higher protection rates than that of the control group. Our antibody level detective results were coordinated with the challenge experiments results, indicating the Δ*ssnA* provided good protective efficacy.

Cell-mediated immunity to vaccination is essential to control infection in the host. In our study, significant lymphoproliferative responses of the immunized mice were detected by MTT assay. Th1 cells are in charge of regulating cellular immune and involved in host defense by producing TNF-α, IL-2, and IFN-γ, while Th2 cells are responsible for coordinating humoral immunity, are involved in host defense by secreting IL-4, IL-5, IL-10, and IL-13 [31]. Our results of the cytokines secretion showed the dominance of Th1 immune response induced by the Δ*ssnA*. Recent study has also demonstrated that *S*. *suis* double mutant vaccine will evoke strong Th1 responses [32].

To successfully colonize the host cell and stimulate immune responses is a necessary condition for good attenuated live vaccine candidate [33]. Our researches indicated that the Δ*ssnA* mutant maybe an appropriate attenuated vaccine candidate for *S*. *suis* on account of its ability to adhere to and invade epithelial cells, decreased virulence, and the ability to induce protective immune response upon infection.

In summary, our results suggest that the Δ*ssnA* mutant is capable of inducing significant humoral and cell-mediated immune, and confers effective protection in CD1 mouse model against SS2 infection. Therefore, Δ*ssnA* could potentially be used for an attenuated live vaccine development and further evaluation will be performed in pig.
